# Supplementary material for: Splice-Junction-Based Mapping of Alternative Isoforms in the Human Proteome
Source: Cell Rep. Author manuscript; Available in PMC 2020 Jan 15. (PMC6961840; doi:10.1016/j.celrep.2019.11.026)
Supplement: 2 [file NIHMS1546469-supplement-2.zip › DF1/PXD006675/AtrialSeptum/AtrialSeptum_11_TTN_VEAEPAEEVTIMEEK.pdf]

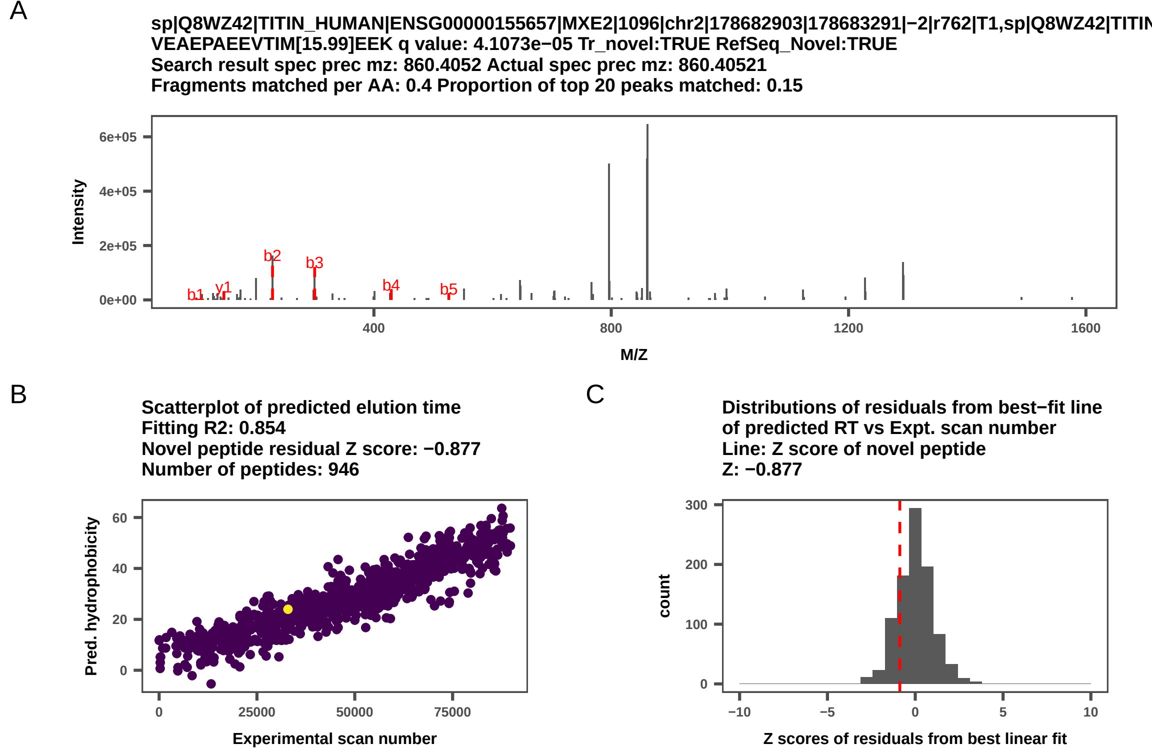

A
sp|Q8WZ42|TITIN_HUMAN|ENSG00000155657|MXE2|1096|chr2|178682903|178683291|−2|r762|T1,sp|Q8WZ42|TITIN
VEAEPAEEVTIM[15.99]EEK q value: 4.1073e−05 Tr_novel:TRUE RefSeq_Novel:TRUE
Search result spec prec mz: 860.4052 Actual spec prec mz: 860.40521
Fragments matched per AA: 0.4 Proportion of top 20 peaks matched: 0.15
Intensity
6e+05
4e+05
2e+05
0e+00
b1
y1
b2
b3
b4
b5
400
800
1200
1600
M/Z
B
Scatterplot of predicted elution time
Fitting R2: 0.854
Novel peptide residual Z score: −0.877
Number of peptides: 946
Pred. hydrophobicity
60
40
20
0
0
25000
50000
75000
Experimental scan number
C
Distributions of residuals from best−fit line
of predicted RT vs Expt. scan number
Line: Z score of novel peptide
Z: −0.877
count
300
200
100
0
−10
−5
0
5
10
Z scores of residuals from best linear fit
